# Development of neurologic diseases in a patient with primate T lymphotropic virus type 1 (PTLV-1)

**DOI:** 10.1186/s12977-016-0290-9

**Published:** 2016-08-12

**Authors:** Yoshimi Enose-Akahata, Breanna Caruso, Benjamin Haner, Emily Charlip, Govind Nair, Raya Massoud, Bridgette J. Billioux, Joan Ohayon, William M. Switzer, Steven Jacobson

**Affiliations:** 1 Viral Immunology Section, Neuroimmunology Branch, National Institute of Neurological Disorders and Stroke, National Institutes of Health, 9000 Rockville Pike, Building 10 Room 5C-103, Bethesda, MD 20892 USA; 2 Translational Neuroradiology Unit, Neuroimmunology Branch, National Institute of Neurological Disorders and Stroke, National Institutes of Health, Bethesda, MD USA; 3 Laboratory Branch, Division of HIV/AIDS, National Center for HIV, Hepatitis, STD, and TB Prevention, Centers for Disease Control and Prevention, Atlanta, GA 30329 USA

**Keywords:** HTLV, PTLV, STLV, HAM/TSP

## Abstract

**Background:**

Virus transmission from various wild and domestic animals contributes to an increased risk of emerging infectious diseases in human populations. HTLV-1 is a human retrovirus associated with acute T-cell leukemia and HTLV-1-associated myelopathy/tropical spastic paraparesis (HAM/TSP). HTLV-1 originated from ancient zoonotic transmission from nonhuman primates, although cases of zoonotic infections continue to occur. Similar to HTLV-1, the simian counterpart, STLV-1, causes chronic infection and leukemia and lymphoma in naturally infected monkeys, and combined are called primate T-lymphotropic viruses (PTLV-1). However, other clinical syndromes typically seen in humans such as a chronic progressive myelopathy have not been observed in nonhuman primates. Little is known about the development of neurologic and inflammatory diseases in human populations infected with STLV-1-like viruses following nonhuman primate exposure.

**Results:**

We performed detailed laboratory analyses on an HTLV-1 seropositive patient with typical HAM/TSP who was born in Liberia and now resides in the United States. Using a novel droplet digital PCR for the detection of the HTLV-1 *tax* gene, the proviral load in PBMC and cerebrospinal fluid cells was 12.98 and 51.68 %, respectively; however, we observed a distinct difference in fluorescence amplitude of the positive droplet population suggesting possible mutations in proviral DNA. A complete PTLV-1 proviral genome was amplified from the patient’s PBMC DNA using an overlapping PCR strategy. Phylogenetic analysis of the envelope and LTR sequences showed the virus was highly related to PTLV-1 from sooty mangabey monkeys (smm) and humans exposed via nonhuman primates in West Africa.

**Conclusions:**

These results demonstrate the patient is infected with a simian variant of PTLV-1, suggesting for the first time that PTLV-1smm infection in humans may be associated with a chronic progressive neurologic disease.

## Background

Virus transmission from various wild and domestic animals has contributed to the increased risk of emerging infectious diseases in human populations. Several recent endemics, such as avian flu, Human immunodeficiency virus (HIV) and ebola, originated from wild animals and which often are asymptomatic but which might induce severe diseases and a pandemic threat in humans [1]. Nonhuman primates (NHP) can be sources of viruses that infect humans and are well-characterized in the natural host and as animal models for some retroviruses [2]. Human T-cell lymphotropic virus type 1 (HTLV-1) is also known to originate from cross-species infection with simian counterparts, simian T-cell lymphotropic virus type 1 (STLV-1) [3, 4]. The majority of HTLV-1 infections remain asymptomatic, but small subsets of infected individuals develop a disease associated with the virus, such as adult T-cell leukemia (ATL), HTLV-1 associated myelopathy/tropical spastic paraparesis (HAM/TSP), and other inflammatory diseases [5–7]. HTLVs and STLVs constitute the primate T-lymphotropic viruses (PTLV) and share some common epidemiological and biological features [4]. Phylogenetic analysis showed that HTLV-1 contains at least seven major subtypes (HTLV-1a to g) [4, 8]. Most humans are infected with the globally distributed HTLV-1 cosmopolitan subtype a which is the only subtype known to be human-restricted and associated with HAM/TSP and ATL. In contrast, the remaining six subtypes closely cluster with STLV-1 strains, of which, five human subtypes together with simian strains are all found in central Africa [4, 9]. Recent epidemiological studies showed that NHP hunters in Cameroon and the Ivory Coast were infected with viruses closely related to STLV-1 strains circulating among local NHPs [9–12], suggesting that, in Africa, recent or ongoing interspecies transmission between simians and humans might likely occurs by severe bites of NHPs and during the collection and consumption of NHP bushmeat [11, 12]. STLV-1 infects a wide variety of Old World primate species of African and Asian origin [2]. Similar to HTLV-1, most STLV-1-infected monkeys remain asymptomatic, but only a small subset of monkeys develops STLV-associated lymphoma/leukemia that shares clinical and pathological features with ATL in humans [13, 14]. However, other clinical syndromes typically seen in humans, such as HAM/TSP, have not been reported in NHPs [6, 7]. Moreover, little is known about the development of neurologic and inflammatory diseases in human populations infected with STLV-1-like viruses following NHP exposures. The lack of disease association in STLV-1-infected NHPs and human likely results from the absence of long term systematic follow-up of these subjects as retroviral disease can take decades to present. Therefore, STLV-1 that cross the species barrier to humans and cause virus-associated neurologic and inflammatory diseases after chronic infection would be of significant public health interest.

Here we demonstrate the detection and sequencing of the complete PTLV-1 genome obtained from a patient with neurologic disease consistent with HAM/TSP. Phylogenetic analysis showed the virus closely clustered with STLV-1 from NHPs in West Africa. These results demonstrate that the patient is infected with the simian variant of PTLV-1, suggesting for the first time that infection of a PTLV-1 strain, clustered with STLV-1 strains from sooty mangabey monkeys, is associated with a chronic, inflammatory, progressive neurologic disease in humans.

## Results

### Clinical follow-up

**Fig. 1:**
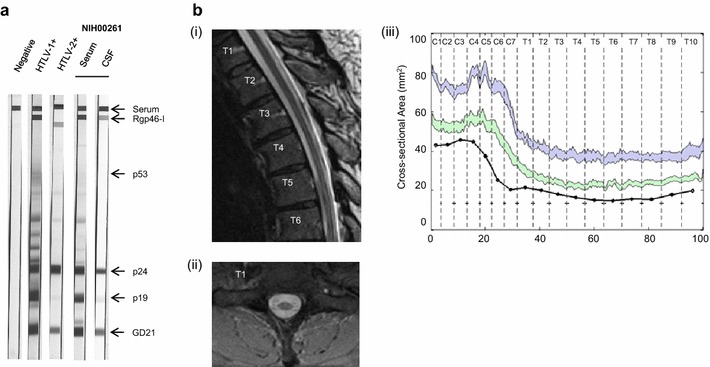
Characteristics of a patient with HAM/TSP (NIH00261). **a** Detection of HTLV-1-specific antibody responses in serum and CSF of patient NIH00261 by Western blot testing. **b** MRI analysis of patient NIH00261. MRI T2-weighted imaging of the patient’s thoracic cord depicting atrophy of the thoracic spinal cord, with a sagittal view of the upper part of the thoracic spine (**i**) and an axial image at level T1 (**ii**). **iii** Profile of the cross-sectional area along the length of the spinal cord in the patient (*black line*). *Shaded region* represent 5 standard errors of the mean in normal healthy donors (NDs) (*blue*, n = 10) and HAM/TSP patients (*green*, n = 10)

Patient NIH00261 was a 65-year old, African male who was originally from Monrovia, Liberia and immigrated to the United State (U.S.) in the 1980s. He was diagnosed with HAM/TSP for approximately 20 years. On examination, he was found to have spasticity and increased reflexes in the lower extremities, moderate lower extremity weakness, a spastic adductor gait, and decreased sensation to vibration in the lower extremities. His disability score was 6.5 in the expanded disability status scale (EDSS) and 16 in the Instituto de Pesquisa Clinica Evandro Chagas disability scale (IPEC). Both serum and CSF from the patient were reactive to HTLV-1 proteins including envelope (Env; rgp46-1 and GD21) and Gag (p19 and p24) (Fig. 1a). Other causes of chronic myelopathy were excluded: laboratory testing for Lyme antibody, B12, folate, copper, rapid plasma reagin, HIV-1 and -2, and rheumatology panel were all negative or within normal limits; cerebrospinal fluid (CSF) analysis showed no evidence of malignancy or other infectious etiologies; and magnetic resonance imaging (MRI) showed no evidence of spinal cord compression, tumor, syrinx, or transverse myelitis. The spinal cord MRI of the patient NIH00261 exhibited atrophy of the entire spinal cord as seen in the mid-sagittal (T1–T6) and axial (T1) T2 weighted scan (Fig. 1b-i, b-ii). Based on the atrophy quantification method [15], we compared the spinal cord cross-sectional area of the patient NIH00261 (black line) to those of the other HAM/TSP patients (green) and normal healthy donors (NDs; blue) (Fig. 1b-iii). The result showed that the entire length of the spinal cord was atrophic in the patient NIH00261, consistent with a clinical finding of HAM/TSP [15].

### Immunologic analysis

**Fig. 2:**
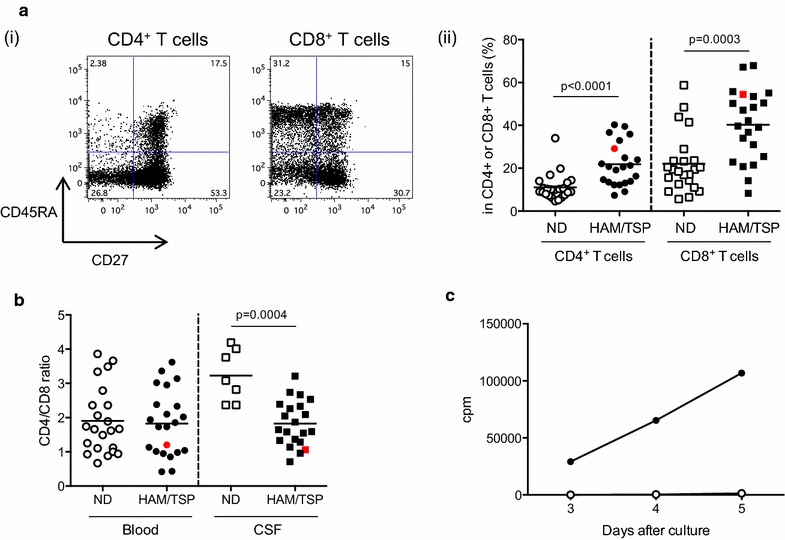
Immunological features of a patient with HAM/TSP (NIH00261). **a** T cell subsets in HAM/TSP patients and NDs. **i** Representative dot plot of CD27 and CD45RA expression on peripheral blood CD4^+^ and CD8^+^ T cells of patient NIH00261. **ii** Frequencies of effector T cell phenotypes in peripheral blood of NDs (n = 22) and HAM/TSP patients (n = 22). *Circles* (*opened* and *closed*) and *squares* (*opened* and *closed*) represent CD4^+^ T cells and CD8^+^ T cells, respectively. Patient NIH00261 results are presented as *red circles* and *squares*. **b** CD4/CD8 T cell ratio in peripheral bloods (*circles*) and CSF (*squares*) of NDs (*opened*; n = 22 in blood and n = 7 in CSF) and HAM/TSP patients (*closed*; n = 22 in blood and n = 21 in CSF). Patient results are shown as *red circles* and *squares*. **c** Spontaneous lymphoproliferation of patient NIH00261 PBMCs. The PBMCs of a ND (*opened circles*) and patient NIH00261 (*closed circles*) were cultured and pulsed with [^3^H] thymidine at 3–5 days. The average cpm from each well in triplicate was plotted

HAM/TSP patients have been reported to show an increased effector T cell phenotype in peripheral blood with increased CD8^+^ T cells in the CSF compared to NDs [16, 17]. Similar to the HAM/TSP group, patient NIH00261 also had increased effector T cell phenotypes, including effector/memory and effector cells in both CD4^+^ and CD8^+^ T cells compared to the control ND group (Fig. 2a-ii). As typical for HAM/TSP, the CD4:CD8 ratio was 1.06 in the patient’s CSF due to a higher frequency of CD8^+^ T cells in his CSF (Fig. 2b). In addition, an established measure of ex vivo T cell activation in HAM/TSP is the well-described observations of increased spontaneous lymphoproliferation [18]. In patient NIH00261, spontaneous lymphoproliferation was also significantly increased linearly during culture compared to ND (Fig. 2c). Collectively, these ex vivo immunological observations are consistent with a diagnosis of HTLV-1-associated inflammatory neurologic disease in patient NIH00261.

### Virologic analysis

**Fig. 3:**
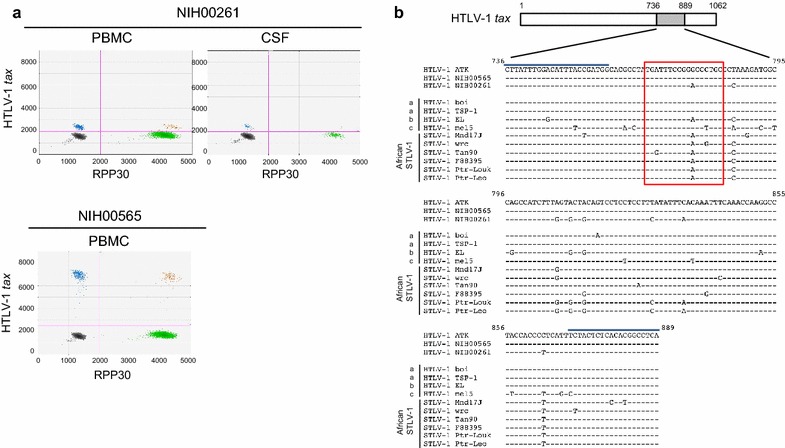
PCR detection of HTLV-1 in a patient with HAM/TSP (NIH00261). **a** Representative two-dimensional ddPCR profiles of the detection of HTLV-1 *tax* sequences in HAM/TSP patients; PBMCs and CSF DNA of patient NIH00261 with divergent HTLV-1 and PBMCs DNA of patient NIH00565 with prototype HTLV-1. HAM/TSP (NIH00565) is a typical HAM/TSP patient evaluated at the NIH. *Y*-axis shows detection of *tax* sequences normalized by detection of human ribonuclease P protein subunit 30 (RPP30) on the *x*-axis. **b** Alignment of HTLV-1 *tax* sequences generated by ddPCR in case (NIH00261) and reference HAM/TSP patients (NIH00565) along with a prototypic HTLV-1 subtype a (ATK). The other prototypic HTLV-1 subtype a variants (boi and TSP-1), subtype b (EL), subtype c (mel5) and African STLV-1 strains (Mnd17J, wrc, Tan90, F88395, Ptr-Loukoum and Ptr-Leo) are also used for comparison. The *tax* region amplified by ddPCR (nt 736-889) is highlighted on the schematic representation of an HTLV-1 *tax* gene (1062 bp; NCBI Gene ID 14191938). Location of primers and probe are *underlined* and *boxed*, respectively

Droplet digital PCR (ddPCR) was recently shown to be a precise and reliable method for HTLV-1 proviral DNA load quantification in samples with low amounts of nucleic acids and for detection of viral mutants in the target gene sequence [19]. Using ddPCR, the proviral load for patient NIH00261 was determined to be 12.98 and 51.68 % in peripheral blood mononuclear cells (PBMCs) and CSF cells, respectively (Fig. 3a). As previously reported [20], higher HTLV-1 proviral loads in CSF cells compared to PBMC also supports the diagnosis of HAM/TSP in patient NIH00261. It has been demonstrated that increased levels of HTLV-1 in CSF cells can differentiate HAM/TSP from other neurologic disease (such as patients with multiple sclerosis infected with HTLV-1) and a concomitant HTLV-1 infection [20]. While there was clear PCR reactivity in both CSF cells and PBMC specimens from patient NIH00261 (Fig. 3a), unexpectedly, the fluorescence amplitude of *tax* sequence was lower in patient NIH00261 compared to a typical HAM/TSP patient NIH00565 (HTLV-1 proviral load; 17.55 %) (Fig. 3a). CSF cells from patient NIH00261 also showed the lower fluorescence amplitudes for detection of the *tax* sequence, which was identical to that observed in the PBMCs of this patient (Fig. 3a). Since fluorescence amplitude reflects the binding affinity of the primers and probe to the target DNA, these results suggested that patient NIH00261 might be infected with an HTLV-1 strain with potential mutations in both peripheral blood and CSF that are different from prototype HTLV-1.

To confirm the presence of genetic mutations in the HTLV-1 from patient NIH00261, we analyzed the *tax* sequences generated with the ddPCR primers (154-bp) from the PBMC of this patient. Sequence analysis demonstrated eight point mutations including one in the probe-binding region of the HTLV-1 from patient NIH00261 compared to a reference sequence from a prototypic variant of HTLV-1 cosmopolitan subtype a (HTLV-1 ATK; Fig. 3b), which is most prevalent in the U.S. and the sequence variability within these strains are very low [8]. These mutations in patient NIH00261 were not observed in the sequence from a HAM/TSP patient NIH00565 as well as the other HTLV-1 cosmopolitan subtype a variants (boi and TSP-1). Interestingly, the *tax* sequence from patient NIH00261 seemed to have similar variations with some African STLV-1 strains (Fig. 3b). Therefore, we generated the complete HTLV-1 genome present in patient NIH00261 by PCR-amplification from PBMC DNA of nine overlapping subgenomic fragments. The complete genome of HTLV-1_NIH00261_ is 9035-bp in length. The overall genomic organization of HTLV-1_NIH00261_ is similar to other PTLV-1, including the presence of intact structural, replication, and regulatory genes (*gag*, protease (*pro*), polymerase (*pol*), *env*, *tax*, *rex, p12I,* and *p30*).

### Phylogenetic analysis of PTLV-1

BLAST analysis identified HTLV-1 and STLV-1 LTR and *env* sequences from Cote d’Ivoire (IC) as having the greatest nucleotide identity to HTLV-1_NIH00261_ [10]. The HTLV-1_NIH00261_ LTR sequence (756-bp) is 99.8 % identical to HTLV-1_Gah050_IC (575-bp) and HTLV-1_Kei005_IC (551-bp) and shared 97.7 % identity with HTLV-1_Pau009_IC and about 98.5 % with STLV-1_Cat_IC (strains 487, 753, 754; 549-bp in length each) from wild sooty mangabey monkeys (*Cercocebus atys*). Similarly, the *env* gene (1464-bp) had the highest nucleotide identity (>99 %) to STLV-1 strains from wild mangabeys from Cote d’Ivoire and from captive mangabeys at two U.S. primate centers [10, 21, 22].

**Fig. 4:**
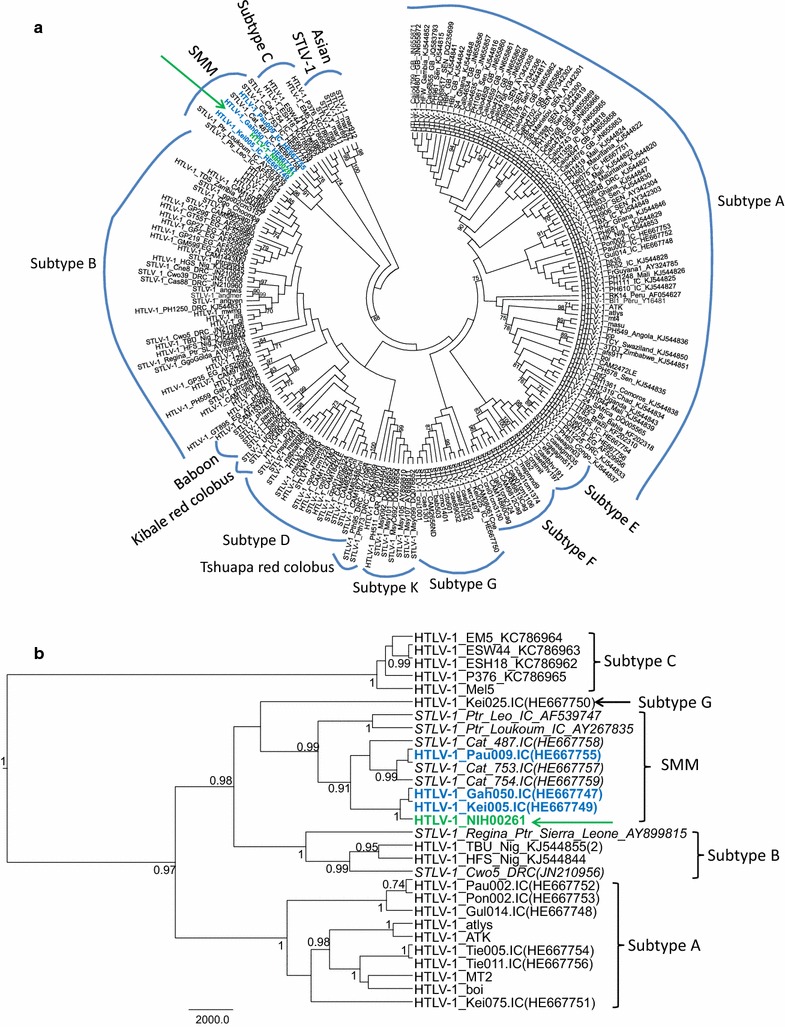
Phylogenetic analysis of PTLV-1 LTR sequences. **a** Maximum likelihood (ML) tree inferred using 207 HTLV-1 and STLV-1 taxa and an LTR alignment of 732 positions. Node support determined using 1000 nonparametric bootstraps. Only bootstrap values >60 are shown. **b** Bayesian-inferred tree using a subset of LTR sequences identified from the ML analysis as having high genetic identify to HTLV-1_NIH00261_ and using selected West African, cosmopolitan, and Australomelanesian reference HTLV-1s for a total of 29 taxa. Posterior probabilities ≥0.7 are shown at nodes. *Scale bar* is in unit of time relative to the mean substitution rate used for the analysis. HTLV-1_NIH00261_ is shown in *green text* and with a *green arrow*. HTLV-1 from three Africans infected with STLV-1 from sooty mangabey monkeys are shown in *blue text*

To further investigate these genetic relationships, we performed detailed phylogenetic analyses using ML and Bayesian inference using both LTR and *env* datasets. Both methods confirmed that the HTLV-1_NIH00261_ LTR was closely related with HTLV-1 and STLV-1 sequences found in Cote d’Ivoire with strong statistical support (Fig. 4). In the LTR trees, three of these HTLV-1 strains (HTLV-1_Gah050, HTLV-1_Kei005, and HTLV-1_Pau009) were reported to be from persons residing in villages close to the Taï National Park, Cote d’Ivoire, and who were exposed to NHPs through activities such as bushmeat preparation and consumption [10]. Within this PTLV-1 LTR cluster (PTLV-1smm), five STLV-1 strains, including three STLV-1_Cat (487, 754, and 753) and two STLV-1_Ptr (Loukoum and Leo), have been reported to be isolated from sooty mangabeys and chimpanzees (*Pan troglodytes*; Ptr), respectively, in the Taï National Park, Cote d’Ivoire [10, 23].

**Fig. 5:**
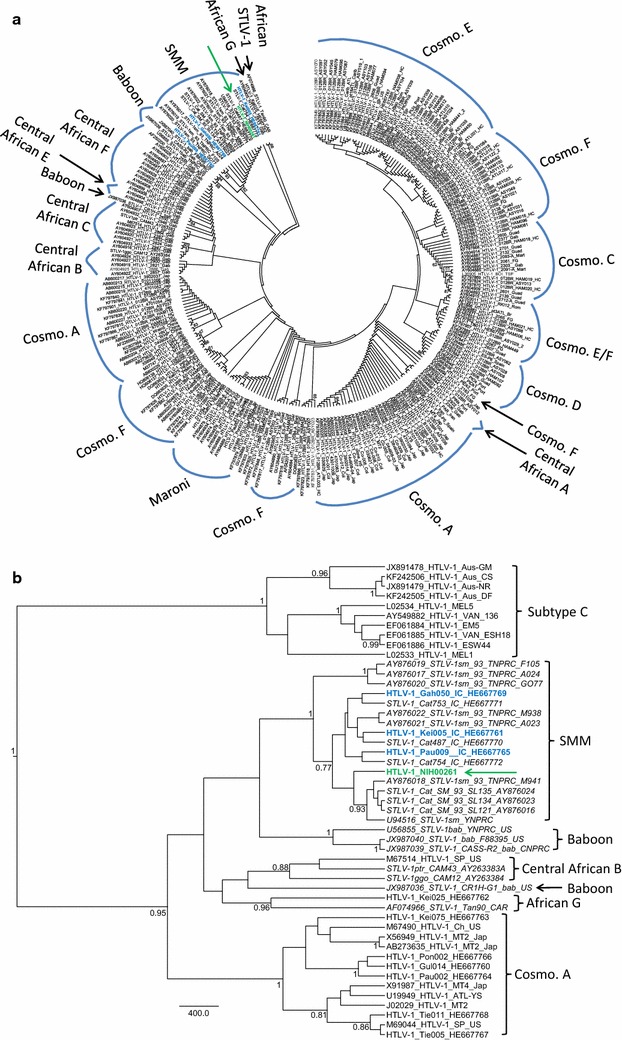
Phylogenetic analysis of PTLV-1 envelope (*env*) sequences. **a** ML tree inferred using 269 HTLV-1 and STLV-1 taxa and an *env* alignment of 426-bp. Node support determined using 1000 nonparametric bootstraps. Only bootstrap values >60 are shown. Cosmo: cosmopolitan. **b** Bayesian inferred tree using a subset of *env* sequences identified from the ML analysis as having high genetic identify to HTLV-1_NIH00261_ and using selected West African, cosmopolitan, and Australomelanesian reference HTLV-1s for a total of 49 taxa. Posterior probabilities ≥0.7 are shown at nodes. *Scale bar* is in unit of time relative to the mean substitution rate used for the analysis. HTLV-1_NIH00261_ is shown in *green text* and with a *green arrow*. HTLV-1 from three Africans infected with STLV-1 from sooty mangabey monkeys are shown in *blue text*

Phylogenetic analysis of the *env* sequences showed that HTLV-1_NIH00261_ also clustered with six STLV-1 isolated from wild sooty mangabeys in Sierra Leone (SL121, SL134 and SL135) and from Cote d’Ivoire (Cat487, Cat753, and Cat754) and with seven STLV-1 from captive sooty mangabeys housed at the Yerkes National Primate Research Center (YNPRC) and the Tulane National Primate Research Center (TNPRC) (Fig. 5) [21, 22]. Since previous studies of STLV-1sm strains at YNPRC and TNPRC suggested that the colonies of sooty mangabeys in the U.S. originated from Sierra Leone [22], phylogenetic analysis of the *env* region demonstrated that HTLV-1_NIH00261_ is highly related with PTLV-1smm strains; HTLV-1 strains in humans exposed via NHP and STLV-1 isolated from sooty mangabeys in West Africa.

## Discussions

PTLVs are deltaretroviruses which by conventional nomenclature are named STLVs when found in NHPs and HTLVs when found in humans, regardless of suspected zoonotic origin [4]. A similar nomenclature is widely used for HIV and their simian counterparts, SIV. Since PTLV genomes are generally stable and their nucleotide substitution rates are significantly lower than those of corresponding lentiviruses, such as HIV and SIV, phylogenetic analysis of PTLV strains from endemic human and simian populations is useful to trace the origin of the virus [24]. Phylogenetic analyses of PTLV-1 corroborated that human and simian strains are interspersed in some subtypes, while others are mainly comprised of human strains without simian counterparts. In Central Africa, STLV-1 strains from chimpanzees or mandrills are indistinguishable from HTLV-1 strains of subtype b or d strains, respectively [25–27]. Human and simian subtype f strains from Gabon and Cameroon are also closely related [26, 28, 29]. However, for the cosmopolitan subtype a, which has spread globally, an STLV-1 counterpart has not yet been identified [8]. Phylogenetic analyses of PTLV-1 therefore have demonstrated that HTLV-1 arose several times from STLV-1 strains by geographically separate interspecies transmissions [8].

In our study, we identified HTLV-1 infection in a male who migrated from Liberia to the U.S. about 30 years ago. HTLV-1 PCR reactivity was initially demonstrated using a novel ddPCR methodology that is a third generation PCR technique that enables the high-precision, direct absolute quantification of nucleic acid target sequences in a given sample [30]. This approach provides wide-ranging applications for quantification of viral DNA [31], detection of more than two target genes (multiplex assay) [32], and importantly has been used to capture rare mutagenesis event [33]. We have reported previously that mutations in regions of HTLV-1 PCR binding probes could be identified using ddPCR [19] and exploited this observation when it was apparent that HAM/TSP patient NIH00261 demonstrated a lower fluorescence amplitude of the HTLV-1 *tax* sequence in both PBMC and CSF when compared to other HAM/TSP patients. This prompted us to obtain a complete, intact viral genomic sequence using PBMC DNA from this patient by PCR amplification of overlapping gene regions for further genetic characterization.

Phylogenetic characterization of two subgenomic regions, LTR and *env*, showed that the virus in this individual was highly similar to PTLV-1smm strains; STLV-1sm found in sooty mangabey monkeys and in persons from West Africa with a history of NHP exposure, including preparing and consuming NHP bushmeat, and who were infected with STLV-1sm from sooty mangabeys living nearby in the Tai National Forest [10]. These results suggest that our patient is also infected with a PTLV-1smm virus and thus raises questions about the origin of his infection. Patient NIH00261 had at least three possible risk factors for PTLV-1 transmission: a blood transfusion from his father, mother-to-child transmission and sexual contacts (with multiple wives). It remains unknown if the PTLV-1 infection occurred through intrafamilial transmission because HTLV seropositivity in his parent and spouses are unknown. Although the exact route of viral transmission is not clear in the patient, it possible that all potential risks likely happened to the patient while he still lived in Liberia. In the U.S., HTLV-1 prevalence is about 0.03 % which consists almost entirely of the cosmopolitan subtype a [8]. Although direct contact between STLV-1-infected NHPs and human is absent or very rare in the U.S. [34], increased global travel and immigration have contributed to the increased risk of virus transmission from various wild and domestic animals in human populations. For example, it has recently been reported that a new strain of HIV-2 was isolated from an immunodeficient patient in New Jersey, which clustered with SIV strains from sooty mangabeys in Sierra Leone from which the patient immigrated [35]. Our patient immigrated to the U.S. in the 1980s and was confirmed to have anti-HTLV-1 antibody responses and HTLV-1 infection in 1998. At that time, since there was no concern for any other PTLV-1 other than HTLV-1 in patient NIH00261, comparative viral genomic analysis was not performed and there was no precise screening method to distinguish between HTLV-1 and STLV-1 infection. The novel ddPCR assay used in our study can simultaneously determine viral loads and detect genetic polymorphism that can be further evaluated by sequence analysis [19]. Our finding also highlights that additional analysis of HTLV-1-infection is needed in persons where NHPs are endemic, or in infected persons migrating from those areas to non-endemic regions to fully understand the prevalence and spread of these PTLV-1 infections closely related to STLV-1 from NHPs.

Similar to HTLV-1, STLV-1 causes chronic infection and leukemia and lymphoma in naturally infected monkeys [13]. Although some clinical signs such as rash and bladder dysfunction have been reported in pig-tailed macaques infected with STLV-1sm [36], to date, cases of other clinical syndromes, such as a chronic progressive myelopathy, have not been reported in NHPs. In human, several HAM/TSP cases have been reported in Africa where HTLV-1 strains cluster with STLV-1 strains [4, 37]. Especially in Central Africa, high frequency of HAM/TSP cases have been reported in northern Zaire concomitant with a high HTLV prevalence in the population [37–40], and most of the Central African strains clustered into HTLV-1 subtype b which clusters with STLV-1 strains from chimpanzee [4, 8]. These results suggested that HAM/TSP might be also caused by HTLV-1 closely related to STLV-1 strains from NHPs, but it is still not clear about the clinical and epidemiological data on HAM/TSP in Africa, immigrants from Africa and humans infected with PTLV-1 closely related to STLV-1 strains following NHP exposure. Patient NIH00261 has all the clinical characteristics of HAM/TSP associated with typical virological and immunological features, including increased proviral load in CSF, spontaneous proliferation, effector T cell phenotypes and CD8^+^ T cell infiltration into the central nervous system [16–18, 20]. Therefore, since it is well established that specific viral genetic mutations in HTLV-1 are not associated with the development of HAM/TSP [41, 42], our results are the first to document that an PTLV-1smm infection in humans is associated with a chronic, inflammatory, progressive neurologic disease. To further explore an association of disease development with this specific strain or PTLV-1 infection from NHPs, systematic epidemiologic studies of PTLV-1 infections in human populations will further improve our knowledge of the pathogenic potential of PTLVs. Such studies will also facilitate determining if this HTLV-1 variant is also spreading in humans.

## Conclusions

We provide evidence that PTLV-1smm transmission to a human is associated with a chronic progressive neurologic disease. Further studies of PTLVs are thus essential for understanding the causes of virus-associated neurologic and inflammatory diseases after chronic infection.

## Methods

### Case history

Patient NIH00261 was a 65-year old, African male who was diagnosed with HAM/TSP in 1998. He was originally from Monrovia, Liberia and immigrated to the U.S. in the 1980s. His symptoms started in the early 1990s with the sub-acute onset of difficulty walking and neurogenic bladder symptoms, which slowly progressed over the ensuing years to the point of needing a cane with ambulation in 1996, and a walker in 2000. He also had lower extremity spasms, occasional paresthesias and sensory loss in his lower extremities, as well as chronic constipation. He was diagnosed in the setting of a work-up for a lower extremity deep vein thrombosis that he developed in 1997. He was seen at the National Institutes of Health (NIH) for the first time in 1998 for evaluation, where he was confirmed to have anti-HTLV-1 antibody responses by Western blot and HTLV-1 DNA by PCR and had a full work-up to exclude other causes for myelopathy. On examination, he was found to have spasticity and increased reflexes in the lower extremities, moderate lower extremity weakness, a spastic adductor gait, and decreased sensation to vibration in the lower extremities. He was lost to follow up until 2012, when he returned for evaluation at the NIH. Over this time period, he had continued slow progression of his neurologic symptoms and did not receive any medical treatments. The patient had a transfusion from his father at the age of 11 while he still lived in Liberia; the HTLV-1 serostatus of his parent and spouses (multiple wives) were unknown; he denied any IV drug use, and while he admitted to eating bushmeat while living in Liberia, he denied ever preparing it or coming into close contact with living NHPs.

### Magnetic resonance imaging (MRI) analysis

MRI of the cervical and thoracolumbar spinal cords was done as previously reported [15]. Briefly, MRI was performed on a 3T Skyra system (Siemens) equipped with a 20-channel head-neck coil and a 16-channel spine-array coil. T1-weighted images were acquired in the cervical spine using 3D-gradient-echo sequences with field-of-view (FOV) = 256 mm, TR = 7.8 ms, TE = 3 ms, 1 mm isotropic resolution, and flip angles of 16°, GRAPPA = 2, for a scan time of about 3.5 min. The sequence was repeated for the thoracic spine, which contains the thoracolumbar cord, by changing the FOV and base resolution to 320 mm in order to cover the larger anatomy while maintaining the 1 mm isotropic resolution. Additional sequences were also used in the cervical and thoracic regions, including short tau inversion recovery (STIR), T2-weighted, T1-MPRAGE, and axial gradient echo.

The cervical and thoracic cords were imaged separately to minimize distortion artifacts at the edge of the imaging region, and 3D distortion correction was used. For analysis, the C- and T-spine images were stitched together using their DICOM information and AFNI’s 3dCalc function and analyzed as a single image. When DICOM information was unable to stitch properly, a few user-placed landmarks near vertebral disks joined the two analyses.

### Specimen preparation

PBMCs were isolated from fresh whole blood using Ficoll-Hypaque (Lonza) centrifugation. Following isolation, PBMCs were cryopreserved in liquid nitrogen or stored as a cell pellet at −80 °C until use. CSF was centrifuged at 1300 rpm for 10 min at 4 °C immediately after collection by lumbar puncture. The CSF supernatant and the cell pellet were collected and stored at −80 °C until use.

### Western blot analysis

Serum and CSF samples were tested for confirmation of HTLV-1/2 antibodies using HTLV Blot 2.4 kit (MP Biomedical) following the manufacturer’s instructions. Serum and CSF were diluted 1:50 for testing.

### Flow cytometry

For analysis of lymphocyte populations in peripheral blood and CSF, EDTA-treated whole blood or CSF cells were stained with antibodies for CD3, CD4, CD8, CD14, CD19, CD27, CD45, CD45RA and CD56 (all from BD Biosciences) and analyzed using a flow cytometer (LSRII; BD Biosciences). Data analysis was performed using FlowJo software (Tree Star). Effector T cell phenotypes including effector/memory and effector phenotypes were defined as CD27^−^CD45RA^−^ and CD27^−^CD45RA^+^, respectively. The Mann–Whitney Test was used to compare effector T cell phenotypes and CD4:CD8 ratio between NDs and HAM/TSP patient using Prism (GraphPad software).

### Lymphoproliferation assay

Measurement of lymphoproliferation was performed as previously described [43]. PBMCs were plated in triplicate at a concentration of 3 × 10^5^ cells/well and were cultured in a 5 % CO_2_ incubator at 37 °C. The cells were pulsed after 3–5 days of culture for 4 h with 1 μCi [^3^H] thymidine. The average cpm from each of the wells was plotted.

### HTLV-1 proviral DNA load

HTLV-1 proviral DNA load was measured using ddPCR (Bio-Rad) as previously described [19]. DNA was extracted from the PBMC and CSF cell pellets using a DNeasy Blood and Tissue kit (Qiagen) according to the manufacturer’s instructions. DNA was digested with the restriction enzyme BamH1 (New England Biolabs) for 30 min at 37 °C, and diluted 1:5 with PCR-certified water. The digested, diluted DNA was mixed with both HTLV-1 *tax* and human ribonuclease P protein subunit 30 (RPP30) primers and probes and Bio-Rad 2× Supermix, and then emulsified with droplet generator oil using a QX-100 droplet generator according to the manufacturer’s instructions (Bio-Rad) [19]. The following primers and probe were used to amplify and detect a 154 base pair region of HTLV-1 *tax*: ddPCR HTLV-1 *tax* F: 5′-CTTATTTGGACATTTACCGATG-3′; ddPCR HTLV-1 *tax* R: 5′**-**TGAGGCCGTGTGAGAGTAGA-3′; ddPCR HTLV-1 *tax* probe: 6FAM-TGATTTCCGGGCCCTGC-MGBNFQ [19]. The droplets were then transferred to a 96-well reaction plate (Eppendorf) and heat-sealed with pierceable sealing foil sheets (Thermo Fisher Scientific). The duplex PCR amplification was performed in this sealed 96-well plate using a GeneAmp 9700 thermocycler (Applied Biosystems) [19]. Following PCR amplification, the 96-well plate was transferred to a QX100 droplet reader (Bio-Rad). For proviral load calculation, QuantaSoft software version 1.3.2.0 (Bio-Rad) was used to quantify the copies/μl of each queried target per well. All samples were tested in duplicate, unless otherwise specified, and proviral load is reported as the average of the two measurements. The proviral load was calculated using the following formula: proviral load = {quantity of HTLV-1 *tax*/(quantity of RPP30/2)} × 100 %.

### Viral genome sequencing

Table 1 lists the primers used to obtain the complete PTLV-1 genome from patient PBMC DNA using PCR to generate nine overlapping fragments. PCR amplification was performed using Platinum^®^ PCR SuperMix High Fidelilty (Life Technologies) as follows: 10 min at 94 °C, 30 cycles consisting of a 30 s denaturation at 94 °C, a 30 s annealing at 55 °C, and a 60 s extension at 68 °C. PCR products were electrophoresed on 1 % agarose gel, then extracted and purified using a QIAquick gel extraction kit (Qiagen). The purified PCR product was ligated with the pCR-4™ TOPO^®^ TA cloning vector (Life Technologies) and transformed into One Shot^®^ Top10 chemically competent *E. coli* (Life Technologies). Plasmids containing each viral DNA insert were purified from transformed E.coli using QIAprep^®^ Miniprep (Qiagen) and sequenced using T3 and T7 primers (Genewiz). The complete HTLV-1_NIH00261_ genome has been assigned the GenBank accession number (KU214243).

**Table 1:** PCR primer pairs used for amplification of overlapping subgenomic PTLV-1 fragments

Fragment no.	Name	Primer sequences 5′–3′
1	Px 23 ACDFN	Fwd: TCATTTCTACTCTCACA
	LTR U5E	Rev: CGCAGTTCAGGAGGCACCACAGGCG
2	LTR400-F	Fwd: CATCCACGCCGGTTGAGTCGC
	Gag1400-R	Rev: GCTGGTGATGGAGGGAAGCTA
3	Gag1350-F	Fwd: CAAAGACCTCCAAGACCTCCT
	Pol2520-R	Rev: TCTAGCCCAAGGACGGCTGGC
4	Pol AG 1	Fwd: GTCGTGATGCCTTACAACAATGCC
	Pol AG 2	Rev: GGGCATGTAGCCAGACAAGTGGCC
5	SK54	Fwd: CTTCACAGTCTCTACTGTGC
	SK111	Rev: GTGGTGAAGCTGCCATCGGGTTTT
6	SK110	Fwd: CCCTACAATCCAACCAGCTCAG
	SG453	Rev: GCGGGATCCTAGGGTGGGAACAG
7	ENV 1	Fwd: TCAAGCTATAGTCTCCTCCCCCTG
	ENV 2	Rev: GGGAGGTGTCGTAGCTGACGGAGG
8	Env3-F	Fwd: ACAAACTGGAATCACCCTTGTTGC
	SK44	Rev: GAGCCGATAACGCGTCCATCG
9	SK43	Fwd: CGGATACCCAGTCTACGTGT
	Tax ddPCR-R	Rev: TGAGGCCGTGTGAGAGTAGA

### Sequence analysis

Nucleotide identities were determined using Geneious v8.1.5 and genome open reading frames and structure were determined by MacVector v13.5.32. Since the number of complete PTLV-1 genomes available at GenBank is limited we restricted the phylogenetic analysis to the envelope (*env*) and LTR regions which have the highest representation of HTLV-1 and STLV-1 sequences at GenBank and have demonstrated utility for inferring PTLV-1 evolutionary histories [3, 9–11]. A BLAST search of the GenBank database using the LTR and *env* sequences was done to identify highly related reference sequences for the analysis. Reference sequences were selected based on the BLAST quality scores (E value) and sequence length. Sequences were aligned in MEGA6 using the MUSCLE program [44] followed by manual editing. The best fitting distance model of nucleotide substitution for each alignment was inferred using the maximum likelihood (ML) method with goodness of fit measured by the Bayesian information criterion in MEGA6. The kimura-2 parameter (K2P) nucleotide substitution model with gamma (G) distributed rates gave the best fit to both datasets followed by the Hasegawa–Kishino–Yano (HKY) + G model. ML phylogenies were inferred using the K2P model implemented in MEGA6. 1000 nonparametric bootstrap replicates were used to assess the strength of the inferred relationships and ML tree topologies. ML trees were visualized and edited with the Tree Explorer program in MEGA6. Bayesian inference was also performed using the program BEAST v1.8.2 on a subset of sequences identified as highly phylogenetically related to HTLV-1_NIH00216_ from the ML analysis using HKY + G model [45]. The smaller datasets were utilized to minimize computational algorithm complexity and to maximize the alignment length and phylogenetic signal in the data. The selected sequences were re-aligned using MUSCLE and for the LTR resulted in a slightly longer alignment of 763 positions compared to 732 positions with the larger dataset. The length of the smaller *env* dataset was unchanged due to the shorter length of some GenBank reference sequences included in the analysis. For both alignments the Australomelanesian HTLV-1 sequences were used as outgroups and we also included some cosmopolitan HTLV-1a for comparison. The following parameters were used for the BEAST analysis: a lognormal molecular clock, the HKY + G nucleotide model, a birth–death process tree prior, and a normal distribution of substitution rate (LTR, mean 2.67E−06, s.d. 4.5E−07; *env*, mean 4.01E−06, s.d. 7.45E−06) previously inferred for HTLV-1 vertical transmission [24]. Two independent 100 million Markov chain Monte Carlo (MCMC) generations were done for each viral region with sampling every 10,000th generation. Convergence of the MCMC was assessed by calculating the effective sampling size (ESS) of the runs using the program Tracer v1.6.0 (http://beast.bio.ed.ac.uk/Tracer). All parameter estimates showed significant ESSs >4000 indicating sufficient mixing. The tree with the maximum product of the posterior clade probabilities (maximum clade credibility tree) was chosen from the posterior distribution of 9001 sampled trees after burning in the first 1000 sampled trees with the program TreeAnnotator version 1.8.2 [45].
